# Effect of main family caregiver’s anxiety and depression on mortality of patients with moderate-severe stroke

**DOI:** 10.1038/s41598-021-81596-8

**Published:** 2021-02-02

**Authors:** Jianting Zhao, Zhilei Zeng, Jianping Yu, Jianhua Xu, Ping Chen, Yujun Chen, Jing Li, Yulong Ma

**Affiliations:** 1 Department of Neurology, Xinxiang Central Hospital, Xinxiang, 453000 Henan China; 2 Department of Neurology, The Second Affiliated Hospital of Zhengzhou University, Zhengzhou, 450014 China; 3 Department of Neurology, The First Affiliated Hospital of Chengdu Medical College, Chengdu, 610500 Sichuan China; 4 Department of Neurology, Fengqiuxian People’s Hospital of Henan Province, Xinxiang, 453300 Henan China

**Keywords:** Cardiovascular diseases, Risk factors

## Abstract

Anxiety and depression are common mental illness in stroke caregivers, resulting in significant stress to the emotion health of caregivers. Caregivers’ emotion can seriously affect the recovery rate of stroke patient, therefore, how to control and affect the caregivers’ anxiety and depression is of great importance. Here three multiple centers observation and validation study were performed to screen out the risk factors for development of anxiety and depression in main family caregiver, and the effect of anxiety and depression of family caregivers on 6-month mortality of patients with moderate-severe stroke. The severity of the stroke, the duration of care time and the medical payment associated with increased risk of anxiety and depression. Anxiety and depression of main family caregivers are associated with increased risk 6-month mortality of patients with moderate-severe stroke. Therefore, the support provided to the family caregivers might have positive effect on prognosis of the patients with stroke.

## Background

Stroke is the dominant leading cause of death and disability among Chinese adults for recent 3 decades^1^. Although great progress has been made in the treatment of stroke in recent years, numerous stroke patients had moderate-severe disability when discharged from the hospital^2–4^. Hence, the main caregivers must have to take care of the patients' physical and daily activities, including communication and emotional support. However, the long-time care process may cause great psychological pressure on the family caregivers, while it also brings a heavy burden, the anxiety and depression introduced silently^5,6^.

Previous studies disclosed that the high incidence rate of anxiety and depression occurred among family caregivers^6–10^. The prevalence of anxiety and depressive symptoms for main caregivers of patients with stroke have been reported to 30%-45% and 20–50%, respectively^5,11,12^. Many studies suggested that the anxiety or depressive symptoms of caregivers have a negative impact on caregivers’ socioeconomic status, physical health and quality of life^13–15^. When main caregivers are in a state of anxiety or depression, they may not provide valuable support for stroke survivors any more, which may reduce the efficiency of the rehabilitation of patients and cause adverse effects on the prognosis of disease^16^.

China is a developing country, traditionally the most stroke patients live at home after discharge. Moreover, most of family caregivers were not received any profession training to care the stroke survivor. This informal care often takes a big amount of time and effort, which might result in overwhelming feelings. Previous studies demonstrated that anxiety or depressed caregivers might impair the quality of life and associated with decreased physical function of stroke patients^11,17,18^. There are few studies investigated the rate of anxiety or depression in main family caregivers of patients with moderate-severe stroke, and the association with the mortality of patients.

Therefore, we are to investigate the risk factors for development of anxiety and depression among main family caregivers and the effect of anxiety or depression of family caregivers on 6-month mortality of patients with moderate-severe stroke.

## Methods

### Ethics approval and consent to participate

This study was approved by the Institute of Institutional Review Board and by the Ethics Committee of Xinxiang Central Hospital, The Second Affiliated Hospital of Zhengzhou University, The First Affiliated Hospital of Chengdu Medical College, and the Fengqiuxian People’s Hospital of Henan Province. All experiments were performed in accordance with relevant guidelines and regulations. All participants provided written informed consent.

### Study population

This was a multicenter prospective study, the consecutive patients with neurological symptom due to cerebrovascular disease were collected within 30 days of symptom onset from January 2015 to February 2020. Cerebrovascular disease was diagnosed according to the diagnostic criteria of the 4th National Cerebrovascular Disease (The 4th National Symposium on Cardiovascular Disease of the Chinese Medical Association,1996). Classification of stroke was confirmed by brain computed tomography (CT) scan or magnetic resonance imaging (MRI). The severity of stroke was assessed by using the National Institutes of Health Stroke Scale (NIHSS). Moderate-severe stroke was defined as admission NIHSS ≥ 6. All the patients and their caregivers were paired, the caregiver group comprised family members who lived with the patient by take caring of the patients' physical and all daily activities, including communication, emotional support.

### Inclusive and exclusive criteria

Paired caregivers were recruited to this study when patients were recruited. The main caregiver was defined as a family member who spent a lot of time with the patient, supported the patient physically and mentally, and helped the patient in daily activities, including assisted patient in walking, turning over in bed, feeding, defecating, and bathing. Caregivers were recruited in the study only if they fulfilled all the following criteria: (1) ≥ 18 years old; (2) The main caregivers were a family member of the patient; (3) Caregivers began to care the patients after stroke, and care time was more than 3 months, ≥ 6 h per day. Caregivers were excluded if they fulfilled the following criteria: (1) Any paid caregivers; (2)Caregivers with severe renal failure (estimated glomerular filtration rate < 30 ml/min.1.73 m^2^), severe pulmonary disease, active malignancies, hypoplasia; (3) incomplete investigation due to communication or reading and writing difficulties. (4) a history of mental illness, and (5) alcohol or drug abuse. The physical health status of the caregivers was obtained through the medical history. Liver and kidney function were obtained by physical examination report within the prior 3 months. Paired caregiver who could not provide the physical examination report, liver function, kidney function and blood count were measured within 48 h after patients’ admission.

Patients were recruited if they met the following criteria: (1) Admission for first-ever stroke within 1 month, and modified Rankin Scale (mRS) score was 0 before stroke; (2) NIHSS ≥ 6 and lived at home after discharge. Patients were excluded if they fulfilled the following criteria: (1) Patients with neurological symptom unrelated to cerebrovascular disease; (2)Severe atrioventricular block and supraventricular arrhythmia; (3)Active malignancies, renal failure(estimated glomerular filtration rate < 30 ml/min.1.73 m^2^), severe pulmonary disease, liver failure, or procedural or surgical complications.

### Data collection

Patients’ demographics and clinical characteristics including vascular risk factors (e.g., hypertension, diabetes mellitus, hyperlipidemia, and smoking history) were collected. While, the caregivers’ demographics included age, sex, duration of care time, education level, care time per day > 8 h(h), and patients’ medical payment method were collected as well. All patients’ and caregivers’ the baseline characteristics were collected within 48 h after admission.

The severity of anxiety was assessed by the 14-item Chinese version of Hamilton Anxiety Rating Scale (HARS)^18–20^ in main family caregivers at 1 month after stroke. Each item is scored from 0 to 4 points, the total score is 0–56, and the anxiety level can be divided as follows: < 7 means no anxiety, 7–14 means possible anxiety, 15–21 means certain anxiety, 21–29 means obvious anxiety and > 29 means severe anxiety. Certain anxiety symptoms were defined by a HARS score > 14^20^.

Meanwhile, the severity of depression was assessed by the 17-item Chinese Hamilton Depression Rating Scale (HDRS)^21,22^,each item is scored on a 2 or 4 point scale, and the depression level can be divided as follows: 0–6 points considered no depression, 7–16 as mild depression,17–24 as moderate depression, and > 24 means severe depression. Certain depressive symptoms were defined by a HDRS score > 17.

The Chinese version of the 14-item HARS has been widely used in the Chinese population, indicating good reliability and validity (20). The validity and reliability of the 17-item Chinese HRSD had been proven in previous studies^22^, and interrater reliability was excellent, the item total score correlations were good, and the internal reliability was satisfactory. A neurologist was trained by psychiatrist with certification qualification of China’s National Health Commission in HARS and HDRS assessment in each hospital, who performed HARS and HDRS assessment of caregivers. The interobserver data differences were resolved by consensus.

The patients lived at home on discharge. All patients and caregivers were followed up once a month for a total of 6 months. All patients were followed up by face-to-face or telephone interview, and patient’s information was obtained from hospital medical records.

### Statistical analysis

The data were presented as median values (interquartile range [IQR]), numbers (%), or mean values (± standard deviation). To distinguish the subgroup difference, the Pearson χ^2^ test was used to categorical variables. Besides, student’s test was used to compare normally distributed variables. Mann–Whitney analysis was used to compare the nonnormal distributed variables. Logistic regression analysis was used to determine the association between anxiety or depression of caregivers and 6-month mortality of patients with stroke. The results were expressed as adjusted odds ratios (aOR) with their corresponding 95% confidence intervals (CI). The data was analyzed using SPSS 22 software. *P* < 0.05 was considered statistically significant.

## Results

### Characteristics of the main caregivers

**Figure 1:**
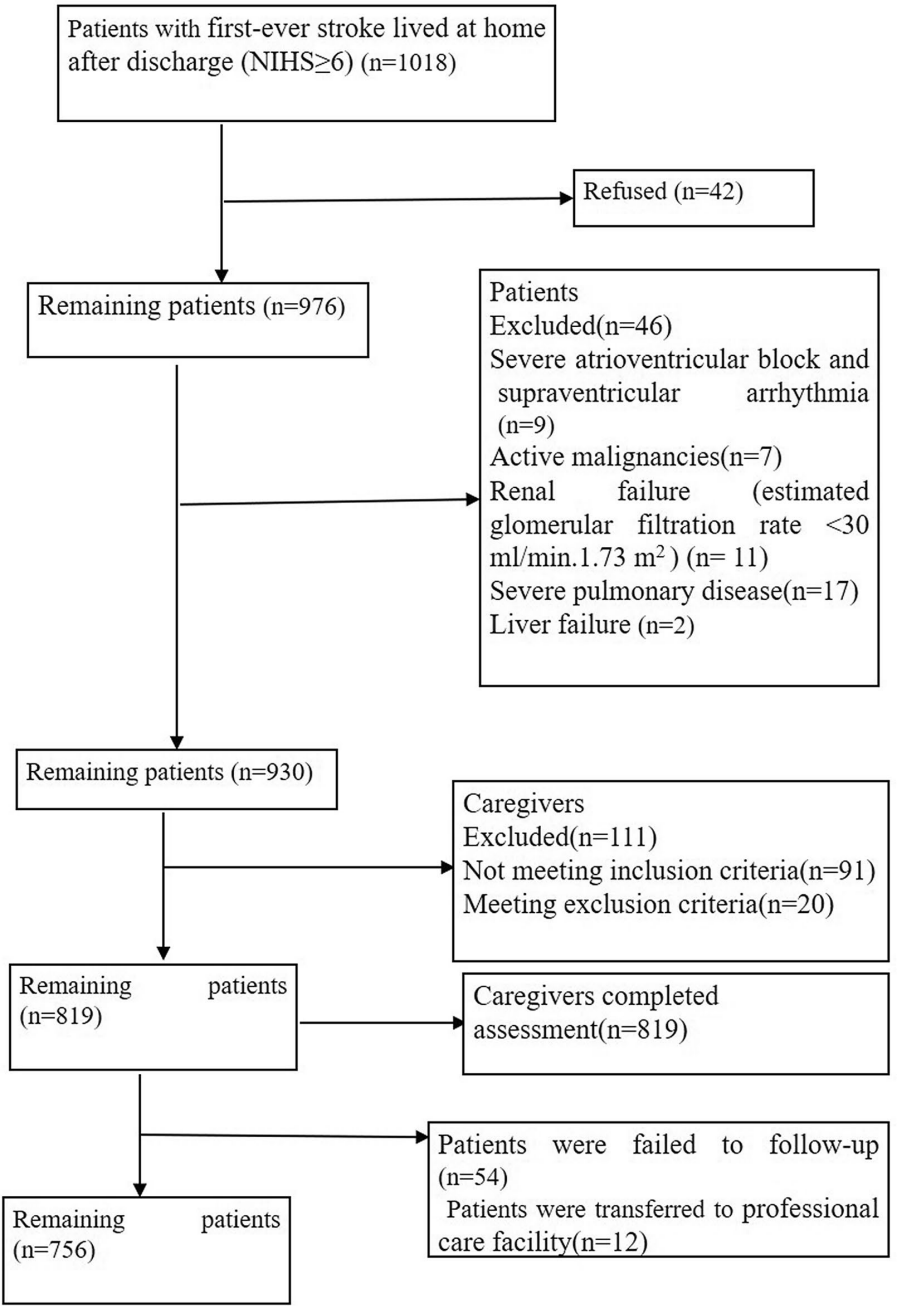
Patient’s and caregiver’s flowchart.

A total of 1018 patients with first-ever stroke lived at home after discharge (NIHS ≥ 6) were included from four multicenter, 42 patients refused to participate in the study, 46 patients met exclusion criteria, 111 paired caregivers were excluded because they met 1 or more of the other exclusion criteria (Fig. 1), the remaining 819 caregivers (249 male; 570 female) were recruited in this study, and the mean age was 55.64 ± 10.09 years (18–88 years). All the main family caregivers were relatives, 512 (62.52%) were spouse of the patients, 290(35.41%) were offspring, and 17(2.08%) were parents. The average care duration was 138.3 ± 28.7 days (93–183 days), caregivers with care time per day > 8 h were 477 (58.2%). The detail basic characteristics of the main family caregivers were shown in Table 1.

**Table 1:** Comparison of baseline characteristics between caregivers with no anxiety and anxiety groups.

	No anxiety group (522)	Anxiety group (297)	OR (95% CI)	P*
Caregivers’ age, y (Mean SD)	54.97±10.09	56.81±10.00		0.014
NIHSS of patients, median (IQR)	8 [6–12]	10 [7–13]		< 0.001
Duration of care time, d (Mean SD)	130.46±25.78	152.08±28.54		< 0.001
Females, n (%)	359 (68.77)	210 (70.71)	1.10 (0.80–1.50)	0.564
Men, n (%)	163 (31.23)	87 (29.29)	1.10 (0.80–1.50)	0.564
Education (high school and above), n (%)	209 (40.04)	123 (41.41)	1.06 (0.79–1.41)	0.700
Care time per day >8 h, n (%)	270 (51.72)	207 (69.70)	2.15 (1.59–2.90)	< 0.001
Relationship to the patients				
Spouse, n (%)	320 (61.30)	192 (64.65)	1.15 (0.86–1.55)	0.342
Other family member, n (%)	202 (38.70)	105 (35.35)	1.15 (0.86–1.55)	0.342
Self–finance, n (%)	122 (23.37)	142 (47.81)	3.00 (2.321–4.07)	< 0.001
Household income, Chinese Yuan (CNY) <5000/month, n (%)	237 (45.40)	144 (48.48)	0.88 (0.66–1.18)	0.395

### The risk factors for anxiety

According to the result of HARS, 297 (36.26%) main family caregivers had anxiety. The average HARS of main family caregivers with anxiety was 21.41 ± 7.80, the average HARS of main family caregivers with no anxiety was 9.18 ± 3.27. Baseline characteristics of caregivers in the no anxiety and anxiety groups were compared (Table 1). The results uncovered that caregivers’ age, NIHSS of patients, duration of care time, care time per day > 8 h and self-finance had effects on the anxiety of main family caregivers, and they presented significant difference (*P* < 0.05).

The anxiety level of caregivers of medical insurance group was lower than that of self-financed group (12.54 ± 7.31 vs 15.88 ± 8.77, *P* < 0.001), and the anxiety level of caregivers with care time per day > 8 h was higher than that of care time per day ≤ 8 h (14.83 ± 8.79 vs 11.91 ± 6.26, *P* < 0.001).Caregivers in anxiety group had older age, higher NIHSS of patients, longer duration of care time, higher percentage of care time per day > 8 h and self-finance than caregivers in no anxiety group(both *P* < 0.05).

To further analyze the risk factors for the anxiety of main family caregivers, multivariable logistic regression was performed, after adjusting for caregivers’ age, NIHSS, duration of care time, sex, education, care time per day > 8 h, self-finance, relationship to the patients, household income, the results showed that NIHSS (aOR, 1.08; 95% CI, 1.04–1.12; *P* = 0.001), duration of care time(aOR, 1.03; 95% CI, 1.02–1.04; < 0.001),care time per day > 8 h (aOR, 1.50; 95% CI, 1.08–2.10; *P* = 0.016), self-finance (aOR, 2.52; 95% CI, 1.80–3.52 *P* < 0.001) were associated with increased risk of anxiety. Among them, self-finance and care time per day > 8 h were the major risk factors for anxiety (Table 2).

**Table 2:** Multivariable Models Showing Association Between baseline risk factors and anxiety.

	OR (95% CI)	P*
NIHSS	1.08 (1.04–1.12)	0.001
Duration of care time	1.03 (1.02–1.04)	< 0.001
Care time (> 8 h)	1.50 (1.08–2.10)	0.016
Self-finance	2.52 (1.80–3.52)	< 0.001

*Multivariable adjusted for caregiver’s age, NIHSS, duration of care time, sex, education, care time per day > 8 h, self-finance, relationship to the patients, household income.

### The risk factors of depression

According to the result of HDRS, 192(23.44%) main family caregivers had depression. The average HDRS of main family caregivers with depression was 23.11 ± 6.64, the average HDRS of main family caregivers with no depression was 8.57 ± 5.03. Baseline characteristics of caregivers in the no depression and depression groups were compared (Table 3). The results showed that caregivers’ age, NIHSS of patients, duration of care time, care time per day > 8 h and self-finance had effects on the depression, and the difference among two groups was statistically significant (*P* < 0.05). The depression level of caregivers of medical insurance group was lower than that of self-financed group (11.48 ± 7.89 vs 13.03 ± 8.81, *P* = 0.012), and the depression level of caregivers with care time per day > 8 h was higher than that of care time per day ≤ 8 h (13.04 ± 8.92 vs 10.51 ± 6.88, *P* < 0.001).Caregivers in depression group had older caregivers’ age, higher NIHSS, longer duration care time, higher percentage of care time per day > 8 h and self-finance than caregivers in no depression group (both *P* < 0.05).

**Table 3:** Comparison of baseline characteristics between caregivers with no depression and depression groups.

	No depression group (627)	depression group (192)	OR (95% CI)	P*
Caregivers’ age, y (Mean SD)	54.40 ± 9.97	59.70 ± 9.44		< 0.001
NIHSS of patients, median (IQR)	9^7–12^	10^7–14^		< 0.001
Duration of care time, d (Mean SD)	133.49 ± 26.66	154.03 ± 29.74		< 0.001
Females, n (%)	439 (70.02)	130 (67.71)	0.90 (0.63–1.27)	0.544
Men, n (%)	188 (29.98)	62 (32.29)	0.90 (0.63–1.27)	0.544
Education (high school and above), n (%)	256 (40.83)	76 (39.58)	0.95 (0.68–1.32)	0.758
Care time per day > 8 h, n (%)	338 (53.91)	139 (72.40)	2.24 (1.58–3.19)	< 0.001
Relationship to the patient				
Spouse, n (%)	397 (63.32)	115 (59.90%)	0.87 (0.62–1.21)	0.391
Other family member, n (%)	230 (36.68)	77 (40.10)	0.87 (0.62–1.21)	0.391
Self-finance, n (%)	175 (27.91)	89 (46.35)	2.23 (1.60–3.11)	< 0.001
Household income, Chinese Yuan (CNY) < 5000/month	299 (47.69)	82 (42.71)	1.26 (0.91–1.74)	0.226

In order to further analyze the risk factors for depression. Multivariate logistic regression was performed (Table 4). The results showed that patient’s NIHSS (aOR, 1.06; 95% CI, 1.02–1.11; *P* = 0.012), duration of care time(aOR, 1.02; 95% CI, 1.02–1.04; *P* < 0.001), caregivers’ age (aOR, 1.06; 95% CI, 1.04–1.08; *P* < 0.001), care time per day > 8 h(aOR, 1.50; 95% CI, 1.02–2.22; *P* = 0.039),self-finance (aOR, 1.98; 95% CI, 1.39–2.81; *P* = 0.001) were associated with increased risk of depression.

**Table 4:** Multivariable Models Showing Association Between baseline risk factors and depression.

	OR (95% CI)	P*
NIHSS of patients	1.06 (1.02–1.11)	0.012
Duration of care time	1.02 (1.02–1.04)	< 0.001
Caregivers’ age	1.06 (1.04–1.08)	< 0.001
Care time per day > 8 h	1.50 (1.02–2.22)	0.039
Self-finance	1.98 (1.39–2.81)	0.001

*Multivariable adjusted for caregiver’s age, NIHSS, duration of care time, sex, education, care time per day > 8 h, self-finance, relationship to the patients, household income.

### Characteristics of the patients

During the 6-month follow-up period, 54 patients were failed to follow-up, a total of 765 patients were finally analyzed, comprised 50.98% (390/765) women and 49.02% (375/765) men. The mean age was 67.33 ± 10.62 years (40–96 years). Among 765 main family caregivers, 290(37.91%) had anxiety symptoms, 182(23.79%) had depressive symptoms,81 caregivers had anxiety and depressive symptoms. In the study population, 480 patients had a history of hypertension, 310 had a history of diabetes, 458 had a history of hyperlipidemia, 210 patients smoke, 442 patients had current alcohol drinking.

### Univariable models for predictors of death

During the 6-month follow-up period, 77 out of 765(10.07%) patients had died. Baseline characteristics of patients in the survivor and dead groups were compared (Table 5). At baseline, dead group showed significantly older age (70.78 ± 10.88 vs 66.94 ± 10.53, *P* < 0.001), higher NIHSS (12.96 ± 5.18 vs 9.71 ± 4.00,* P* < 0.001), higher percentage of caregivers with anxiety (70.13% vs 34.30%;OR,4.50;95% CI,2.69–7.51; *P* < 0.001) and caregivers with depression (44.16% vs 21.51%;OR,2.89;95% CI, 1.78–4.69; *P* < 0.001), and higher percentage of caregivers with anxiety + depression (24.68% vs 9.01%;OR,3.31;95% CI,1.85–5.91; *P* < 0.001) than patients with survivor group. Compared with survivor group, dead group had a significantly higher HADS score of caregivers (25.97 ± 12.88 vs 12.38 ± 5.91, *P* < 0.001), and higher HRDS score of caregivers (17.86 ± 13.14 vs 11.37 ± 7.38, *P* < 0.001).

**Table 5:** Comparison of baseline characteristics between survival and dead groups.

	Survivor group (688)	Dead group (77)	OR (95% CI)	P*
Age, y (Mean SD)	66.94 ± 10.53	70.78 ± 10.88		0.002
HADS score, (Mean SD)	12.38 ± 5.91	25.97 ± 12.88		< 0.001
HRDS score, (Mean SD)	11.37 ± 7.38	17.86 ± 13.14		< 0.001
NIHSS, median (IQR)	9^7–12^	12^9–16^		< 0.001
Female, n (%)	354 (51.45)	36 (46.75)	0.83 (0.52–1.33)	0.434
Male, n (%)	334 (48.55)	41 (53.25)	0.83 (0.52–1.33)	0.434
BMI ≥ 24 kg/m, n (%)	199 (28.92)	25 (32.47)	1.18 (0.71–1.96)	0.517
Hypertension, patients, n (%)	430 (62.50)	50 (64.94)	1.11 (0.68–1.82)	0.675
Current Smoking, n (%)	190 (27.62)	20 (25.97)	1.06 (0.63–1.79)	0.759
Current alcohol drinking, n (%)	224 (32.56)	18 (23.38)	0.62 (0.36–1.10)	0.100
Diabetes, n (%)	282 (40.99)	28 (36.36)	0.82 (0.51–1.34)	0.433
Hyperlipidemia, n (%)	410 (59.59)	48 (62.34)	1.12 (0.69–1.82)	0.641
Ischemic stroke, n (%)	545 (79.22)	62 (80.52)	0.92 (0.51–1.67)	0.789
Hemorrhagic stroke, n (%)	143 (20.78)	15 (19.48)	0.92 (0.51–1.67)	0.789
Family history of stroke, n (%)	146 (21.22)	18 (23.38)	1.13 (0.65–1.98)	0.662
Nasogastric tube feeding, n (%)	86 (1.25)	12 (15.58)	1.29 (0.67–2.49)	0.442
Indwelling urinary catheter, n (%)	108 (15.70)	16 (20.78)	1.41 (0.78–2.54)	0.251
Anxiety, n (%)	236 (34.30)	54 (70.13)	4.50 (2.69–7.51)	< 0.001
Depression, n (%)	148 (21.51)	34 (44.16)	2.89 (1.78–4.69)	< 0.001
Anxiety + depression, n (%)	62 (9.01)	19 (24.68)	3.31 (1.85–5.91)	< 0.001
Medication use				
Statin, n (%)	321 (46.66)	43 (55.84)	1.45 (0.90–2.32)	0.126
Antihypertensive, n (%)	346 (50.29)	39 (50.65)	1.01 (0.63–1.63)	0.952

### Multivariable Models on the Association between dead and anxiety/depression of caregivers

In unadjusted models, there was an association between the percentage of caregivers with anxiety or depression and 6-month mortality of patients with moderate-severe stroke. In the study, the results showed that 54 (54/290, 19.66%) patients had died in caregivers with anxiety group, which was higher percentage than that in caregivers with no anxiety group (23/475, 4.84%) (*P* < 0.001); and 34(34/182,) patients died in caregivers with depression group, which was higher percentage than that in caregivers with no depression group(43/583, 4.84%) (*P* < 0.001).

To further analyze the association between anxiety and depression of caregivers and 6-month mortality of patients with moderate-severe stroke, the factors associated with 6-month mortality in the univariate analyses (*P* < 0.20) were entered into the multivariate logistic regression analysis[adjusted for age, baseline NIHSS, current alcohol drinking, the percentage of caregivers with anxiety and depressive symptoms/the percentage of caregivers with anxiety + depressive symptoms (or HADS score and HRDS score of caregivers), statin use], the results showed that older age (aOR,1.03;95% CI, 1.01–1.06; *P* = 0.010), higher NIHSS (aOR,1.12;95% CI, 1.06–1.18; *P* < 0.001), the percentage of caregivers with anxiety (aOR,3.57;95% CI, 2.09–6.11; *P* < 0.001), and the percentage of caregivers with depression (aOR,2.20;95% CI, 1.30–3.72; *P* = 0.003) were associated with increased risk of 6-month mortality of patients with moderate-severe stroke (Table 6), when HADS and HRDS score of caregivers were entered into multivariate logistic regression (Model 2), HADS score(aOR,1.19;95% CI, 1.15–1.24; *P* < 0.001) and HRDS score(aOR,1.10;95% CI, 1.07–1.41; *P* < 0.001) of caregivers resulted to be associated with increased risk of 6-month mortality of patients with moderate-severe stroke, and higher NIHSS (aOR,1.12;95% CI, 1.05–1.19; *P* = 0.001) were associated with increased risk of 6-month mortality, when the percentage of caregivers with anxiety + depression were entered into multivariate logistic regression (Model 3), the results showed that older age (aOR,1.04;95% CI, 1.01–1.06; *P* = 0.003), higher NIHSS (aOR,1.16;95% CI, 1.08–1.20; *P* < 0.001), the percentage of caregivers with anxiety (aOR,2.29;95% CI, 1.21–4.33; *P* = 0.011).

**Table 6:** Multivariable Models Showing Association Between risk factors and 6-month mortality.

	OR (95% CI)	P*
Model 1 (the percentage of caregivers with anxiety and depressive symptoms)		
Age	1.03 (1.01–1.06)	0.010
NIHSS	1.12 (1.06–1.18)	< 0.001
Caregivers with anxiety symptoms	3.57 (2.09–6.11)	< 0.001
Caregivers with depressive symptoms	2.20 (1.30–3.72)	0.003
Model 2 (HADS and HRDS score)		
NIHSS	1.12 (1.05–1.19)	0.001
HADS score of caregivers	1.19 (1.15–1.24)	< 0.001
HRDS score of caregivers	1.10 (1.07–1.41)	< 0.001
Model 3 (the percentage of caregivers with anxiety + depressive symptoms)		
Age	1.04 (1.01–1.06)	0.003
NIHSS	1.14 (1.08–1.20)	< 0.001
Caregivers with anxiety + depression symptoms	2.29 (1.21–4.33)	0.011

*Multivariable adjusted for age, baseline NIHSS, current alcohol drinking, the percentage of caregivers with anxiety and depressive symptoms/the percentage of caregivers with anxiety + depressive symptoms (or HADS score and HRDS score of caregivers), statin use.

## Discussion

Based on these results we found that high incidence of anxiety and depression of main family caregivers of patients with moderate-severe stroke, which were consistent with previous studies, the data showed that 297 (36.26%) main family caregivers had anxiety, and 192(23.44%) main family caregivers had depression^5,23,24^, furthermore, we found that anxiety and depression of caregivers were associated with increased risk of 6-month mortality of patients with moderate-severe stroke.

Stroke is leading reason to cause the disability^25–27^. Previous studies revealed that most stroke survivors’ leave hospital with varying degrees of disability, they are often accompanied by limb hemiplegia and other dysfunctions^28–30^. In China, the most of stroke survivors lived at home after discharge, obviously, the huge and invisible mental pression transfer to the caregivers. In this study, the results disclosed that the higher ratio of caregivers was female, they were the spouse of the patients, which was consistent with previous studies^31^, this may be due to the social and cultural lifestyles in China. In past studies showed that there was no difference in physiological distress between female and male caregivers^32,33^, whereas some studies reported higher rates of psychological distress in females^34,35^.

Meanwhile, our resulted demonstrated that the percentage of anxiety and depression was respectively 36.26% and 23.32% in main family caregivers, which was consistent with others^5,11,12^. The results showed that NIHSS, duration of care time, care time per day > 8 h and self-finance had significant predictive effects on the anxiety and depression of main family caregivers, and older caregivers’ age was a risk factor of depression symptoms. It can be considered that the emotional status of the main family caregivers is related to the level of dependence of the patients on assistance and the economic burden. Stroke treatment and rehabilitation costs are very high. Family economic burden is related with the medical payment method^36^. In our study, we also found that the percentage of main family caregivers with anxiety and depression in self-finance families were higher than those of in medical insurance payment families, which suggested that the economic burden of illness is an important risk factor of anxiety and depression for caregivers. The length of care time is a risk factor for development of anxiety and depression in main family caregiver. In this study, duration of care time and care time per day > 8 h were positively correlated with HARS and HDRS scores. Past study also showed that anxiety and depression were more likely to appear with longer care time^37,38^. This could be because the care time are too long every day, occupied too much of the caregivers' personal time and reducing their work, social and entertainment time in largest degree. On the other hand, the caregivers' age is related to depression, the older the caregiver, the higher the risk of depression, this may be because that elder caregivers had limited energy, which may be more likely to lead to fatigue.

There are few studies on the effect of anxiety and depression on mortality of patients with moderate-severe stroke. In our study, we investigated the association between the anxiety and depression of the main family caregivers and the 6-month mortality of patients with moderate-severe stroke. The results demonstrated that anxiety and depression of caregivers were associated with increased risk of 6-month mortality of patients with moderate-severe stroke. Therefore, it may be suggested that any support such as social and psychological leading to relieve anxiety or depression of the caregivers will be helpful to improve the recovery of the patients.

## Conclusions

In conclusion, caregivers’ age, NIHSS of patients, duration of care time, care time per day > 8 h and self-finance are major risk factors for development of anxiety and depression in main family caregiver. The anxiety and depression of main family caregivers have a negative impact on 6-month mortality of patients with moderate-severe stroke. These results indicate that clinical nurses should provide home care guidance, psychological counseling and social support to family caregivers to improve their mental health.

## Limitation

We selected the patients with moderate-severe stroke, while the follow up of their rehabilitation of patients post discharge were failed. Moreover, we did not collect data on caregivers' use of antidepressants and anxiety and depression in patients with stroke, 14-item Chinese HARS and HDRS score are just screening tools, we used HARS and HDRS to assess whether the caregiver had symptoms of anxiety or depression, and should not be used to diagnosis anxiety and depression, we did not analyze the effect of different levels of caregivers’ anxiety or depression on patients' mortality which might have influenced the results.

## Data Availability

The datasets used and/or analyzed during the current study are available from the corresponding author on reasonable request.

## Abbreviations

HARS: 14-Item Chinese Hamilton Anxiety Rating Scale
HDRS: 17-Item Chinese Hamilton Depression Rating Scale
NIHSS: National Institutes of Health Stroke Scale
CI: Confidence interval
M: Mean
OR: Odds ratio
SD: Standard deviation
IQR: Interquartile range
